# Thrombolysis as Part of Acute ST-Segment Elevation Myocardial Infarction (STEMI) Care: Experience From the STEMI Network in Tanzania

**DOI:** 10.7759/cureus.86337

**Published:** 2025-06-19

**Authors:** Nakigunda Jumanne Kiroga, Khuzeima S Khanbhai, Davis Amani, Peter R Kisenge, Tatizo Waane, Tulizo Shemu, George Longopa, Mazen Albaghdadi, Sigfrid Shayo, Yona Gandye, Zahid Khan

**Affiliations:** 1 Cardiology, Jakaya Kikwete Cardiac Institute, Dar es Salaam, TZA; 2 Epidemiology and Statistics, Muhimbili University of Health and Allied Sciences, Dar es Salaam, TZA; 3 Medicine, Univerisity of Central Florida, Orlando, USA; 4 Cardiology, NCH Rooney Heart Institute, Naples, USA; 5 Cardiology, University of South Wales, Pontypridd, GBR; 6 Cardiology, University of Buckingham, London, GBR; 7 Cardiology, Barts Heart Centre, London, GBR

**Keywords:** " "atherosclerotic cardiovascular disease, cardiovascular disease challenges, cardiovascular disease prevention, " "cardiovascular disease risk factors, cardiovascular medicine. hypertensive heart disease. cardio-oncology. cardiac mri, global burden of disease, injuries and risk factors study, ischaemic heart disease (ihd), low- and middle-income countries (lmic), st-segment elevation mi

## Abstract

Background and objective

The burden of ischaemic heart disease (IHD) in low- and middle-income countries (LMIC) is increasing worldwide. A significant proportion of hospitalizations and deaths have been attributed to this condition. Promoting good clinical outcomes in patients with IHD requires timely diagnosis and management using percutaneous coronary intervention (PCI) and recommended pharmacotherapy. Fibrinolytics, combined with antiplatelet and anticoagulant agents, are recommended as initial therapy in resource-limited settings. The regional systems, which aim to provide access to timely percutaneous intervention for patients with ST-elevation myocardial infarction (STEMI), are still in their infancy stages in Tanzania. However, these systems have shown promising results in coordinated patient management. This survey was conducted through a network to gain an understanding of the general context of IHD management. This study aimed to assess the current service availability, infrastructure in place, and the duration of time taken between various referral centers in different zones.

Methodology

An online cross-sectional survey was conducted between May and June 2023, involving medical practitioners from facilities in mainland Tanzania and Zanzibar. Physicians from various regional centers were invited to complete an anonymous online survey about the available STEMI services and their familiarity with the pathway. Information on geography, available expertise, equipment, and medicines at these facilities was collected and analyzed using R software, version 4.3.1. Proportion and frequencies were the primary measures for assessing the capacity and distribution of these facilities. A chi-square test was used to compare these parameters between public and private facilities, with the significance level set at p<0.05. All ethical standards were adhered to, and approval was obtained from the ethics committee.

Results

Among the 66 clinicians from mainland Tanzania and Zanzibar, 36 (54.5%) worked in public facilities, while 30 (45.5%) worked in private facilities. Clinician responses from the coastal regions were obtained from 18 (50%) public facilities and 10 (33%) private facilities, indicating an uneven distribution of thrombolysis and primary angioplasty services in this region. Only a quarter (n=9, 25%) of clinicians from the public and less than half (n=14, 47%) of the clinicians from private facilities reported the availability of thrombolytic medications in their facilities. Moreover, 11 (31%) and seven (23%) clinicians from private and public facilities, respectively, could refer patients for advanced care within the recommended 90 minutes after initial medical contact. Primary angioplasty services were concentrated in the coastal and central regions of the country, with four catheterization laboratories in the Dar es Salaam region (coastal) and one in the Dodoma Region (central).

Conclusions

Facilities offering medical services to patients with myocardial infarction (MI) are unevenly distributed in Tanzania, with a concentration mainly in the coastal region. These facilities have established protocols to care for patients with STEMI. The capacity of facilities is generally low and varies between public and private facilities. Further research is necessary to inform policies, and deliberate strategies and efforts are required to address the prevailing situation.

## Introduction

Cardiovascular disease (CVD) is the leading cause of morbidity and mortality globally [1,2]. The burden of these conditions has significantly increased over the past three decades. According to the Global Burden of Disease, Injuries and Risk Factors Study (GBD), CVD cases (272 million to 523 million), deaths (12.1-18.6 million), and disability adjusted life years (DALYs, 279.8 million to 393.1 million) have increased drastically between 1990 and 2019 [1,2]. The persistently high burden of these diseases is attributed to the prevalence of common risk factors such as hypertension, obesity, tobacco use, physical inactivity, and dietary factors [3]. The changing landscape of these diseases, also referred to as the epidemiological transition, has not been uniform across the globe or within populations, and new patterns are emerging [2-5]. Recent evidence has shown that some specific types of CVD, such as alcohol-induced cardiomyopathy and rheumatic heart disease, peak at a relatively young age. Low- and middle-income countries (LMICs) have been disproportionately affected [6]. Over 80% of premature CVD deaths have been reported in these contexts [7].

Ischemic heart disease (IHD) has remained one of the leading causes of morbidity and mortality attributable to CVDs for several decades, and the burden of this disease has continued to increase steadily since the 1990s [1,7]. According to the GBD study, the prevalence of cases, deaths, and DALYs due to IHD rose from 1990 to 2019, reaching 197 million, 9.14 million, and 182 million, respectively [1,4]. The mainstay strategy to counteract the burden of CVD includes the prevention and management of modifiable risk factors, early screening, diagnosis, and providing timely intervention to reduce morbidity and mortality. Several distinct variables can affect these essential aspects of the management pathway [3,6]. Timely administration of reperfusion therapy is a cornerstone of positive outcomes among patients with ST-elevation myocardial infarction (STEMI), a subcategory of IHD. Percutaneous Coronary Intervention (PCI) is an advanced and superior form of this therapy; however, it is technically complex to perform and costly. As a result, they are not readily available in resource-limited settings [7-11].

In areas with limited resources, pharmacotherapy, which involves a combination of fibrinolytics and anticoagulants, is the recommended option [9-11]. The alternative use of combination pharmacotherapy has shown promising outcomes, including patency in approximately 75% of patients [10]. Provided that the referral systems are functional, timely and accurate execution of pharmacotherapy options ensures myocardial stability and performance before PCI administration at tertiary centers [1,9]. Realizing these outcomes largely depends on fulfilling the following prerequisites: obtaining a thorough medical history, performing clinical examination, and administering appropriate diagnostic tools such as electrocardiography, cardiac markers, and bleeding indices [1,6,11]. Furthermore, the availability of trained staff to perform these tasks can also affect the service provision.

The WHO Global Action Plan for Prevention and Control of Non-Communicable Diseases, among other things, aims to simultaneously reduce premature deaths from NCDs by 25% and ensure that at least 50% of eligible individuals receive drug therapy by 2025 [12,13]. Sustainable Development Goal 3 further emphasizes this point, insisting that authorities must strive to ensure that individuals have access to quality, safe, and affordable medications for NCDs [13]. The supply of cardiovascular medicines, including fibrinolytics, in LMICs has improved in recent years [6,12]. However, their availability remains suboptimal, varying among nations and within countries and regions with different economies regarding access to these medicines [1,6,7,11]. However, affordability remains a significant challenge. Fibrinolytics are expensive, and most insurance schemes do not cover this vital medicine. As a result, very few patients have access to this drug [6,11].

The outcomes of STEMI have improved tremendously in recent years, particularly in high-income countries [14]. Part of this success is credited to well-established STEMI care networks [14,15]. This has enhanced cooperation among facilities and care providers, facilitating proper management, timely referral, linkage, and continuity of care [16]. Several LMICs, such as Tanzania, have adopted a similar approach. In countries like Tanzania, with scarce resources, the STEMI service was recently established. There has been an increasing collaboration between physicians at various centers to share experiences and discuss issues faced by physicians and patients in different centers, benefiting from this pathway. These surveys mainly focus on issues related to the diagnosis, treatment, and referral of patients with suspected or confirmed STEMI. Recently, these initiatives have led experts to conduct an online evaluation assessing available expertise, diagnostics, fibrinolytics, and referral systems for STEMI patients. The findings were used to inform policies and recommend improvements in current practices. This study presents and discusses its findings and implications.

## Materials and methods

Study design and setting

A cross-sectional study based on an online survey was conducted between May and June 2023. It involved participants from health facilities on the Tanzania mainland and Zanzibar who were providing care and treatment to patients with myocardial infarction (MI). This was a quantitative data study, and data were collected from clinicians working across different centres in the country. 

Data collection

A semi-structured questionnaire with closed-ended questions was developed and piloted before deployment. The initial questionnaire was tested on a group of 15 clinicians across different centres and further modified based on the feedback received. These modifications included adding data on the availability of clinical expertise, distance from the specialist center, and making all sections compulsory for successful completion of the survey. This process was divided into two parts. The first part contained questions on the geography and infrastructural capacity of the facility. In contrast, questions on referral systems, available expertise, and availability of thrombolytic medications and angioplasty services formed the second part. The tool was uploaded to Google Forms and then shared with providers through the STEMI online survey group, which included the contact information of clinicians from various hospitals. The forms were configured in such a way that participants could not submit them without completing all questions. The form was structured in a way that required all questions to be answered for the survey to be considered complete.

Data analysis

Data were analyzed using SPSS Statistics software (IBM Corp., Armonk, NY). Frequency and proportion were the primary measures of analysis in this survey, used to determine the distribution of private and public health facilities by zone and type of facility, and to assess the availability of emergency departments, electrocardiograms (ECGs), and thrombolytics. The chi-squared test was used to compare private and public health facilities based on these characteristics. Referral modalities, referral duration, level of available expertise, and types of medications used were assessed using frequency and proportion. The online survey link was shared with clinicians working in both public and private sector hospitals located in different zones to minimize selection bias. 

Ethical considerations

Before enrolment in this survey, it was ensured that all prospective participants had access to detailed information about the study’s design, benefits, and risks in English and Swahili. We ensured the confidentiality of the study participants by not collecting their personal or identifiable data. The study was approved by the Ethical Review Committee of Jakaya Kikwete Cardiac Institute (JKCI), with the approval number AB.123/307/01K/09.

## Results

Sixty-six clinicians from 18 facilities responsible for thrombolysis decisions responded to the survey. These 18 facilities are located across six administrative zones in Tanzania. Thirty-six (54.5%) clinicians from this group worked in public health sector hospitals. The majority (n=18, 50%) were employed in public health sector facilities in the coastal region. In contrast, over a third (n=11, 37%) of private health facility clinicians were located in the northern region. There was a significant difference between private and public health facilities regarding the availability of thrombolysis and primary PCI services for managing patients with MI. In private health systems, 12 (40%) clinicians were employed in zonal hospitals, whereas in public health systems, 10 (28%) clinicians were from regional hospitals and 10 (28%) were from national hospitals. The remaining 34 clinicians were employed in district general hospitals, health centers, and dispensaries that lack primary angioplasty facilities. The availability of thrombolytics differed significantly between the two groups; nearly half (n=14, 47%) of private facilities and only a quarter (n=9, 25%) of public facilities had access to this medication (Table 1).

**Table 1:** General characteristics of the facilities ^*^Fisher’s exact test; Pearson’s Chi-squared test ECG: electrocardiogram

Characteristic	Private (n=30), n (%)	Public (n=36), n (%)	P-value^*^	Chi-square value
Zone			0.3	5.77
Central	2 (6.7)	3 (8.3)		
Coastal	10 (33)	18 (50)		
Lake zone	2 (6.7)	3 (8.3)		
Northern	11 (37)	5 (14)		
Southern highlands	3 (10)	6 (17)		
Zanzibar	2 (6.7)	1 (2.8)		
Facility type			<0.001	29.91
District hospital	5 (17)	9 (25)		
National hospital	0 (0)	10 (28)		
National super specialty hospital	0 (0)	4 (11)		
Others	8 (27)	0 (0)		
Regional referral hospital	5 (17)	10 (28)		
Zonal referral hospital	12 (40)	3 (8.3)		
Emergency department	24 (80)	29 (81)	>0.9	0.003
ECG availability	30 (100)	35 (97)	>0.9	0
Thrombolytic availability	14 (47)	9 (25)	0.036	2.5

**Figure 1:**
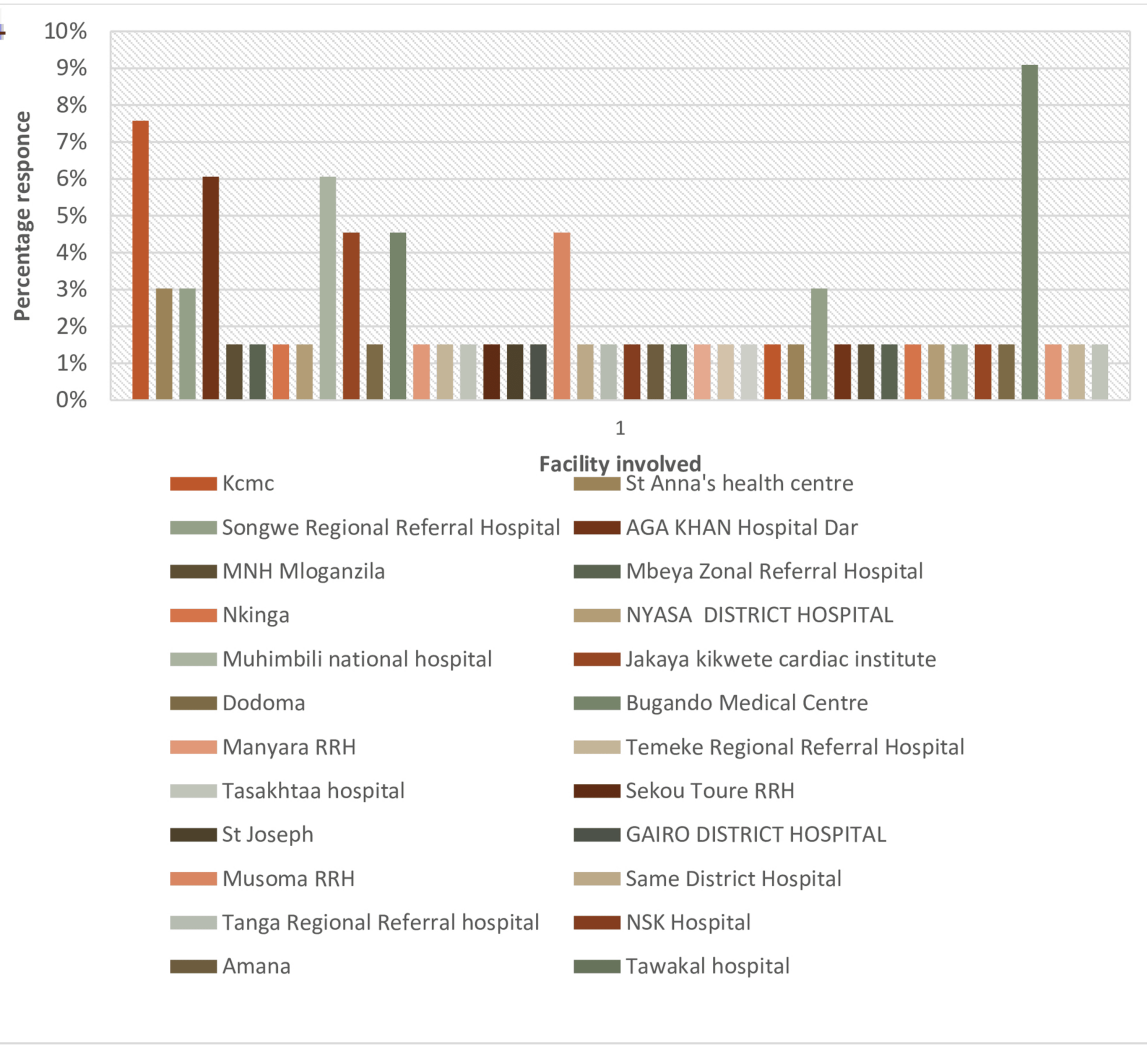
The response rate in various administrative zones/STEMI facilities STEMI: ST-elevation myocardial infarction

**Figure 2:**
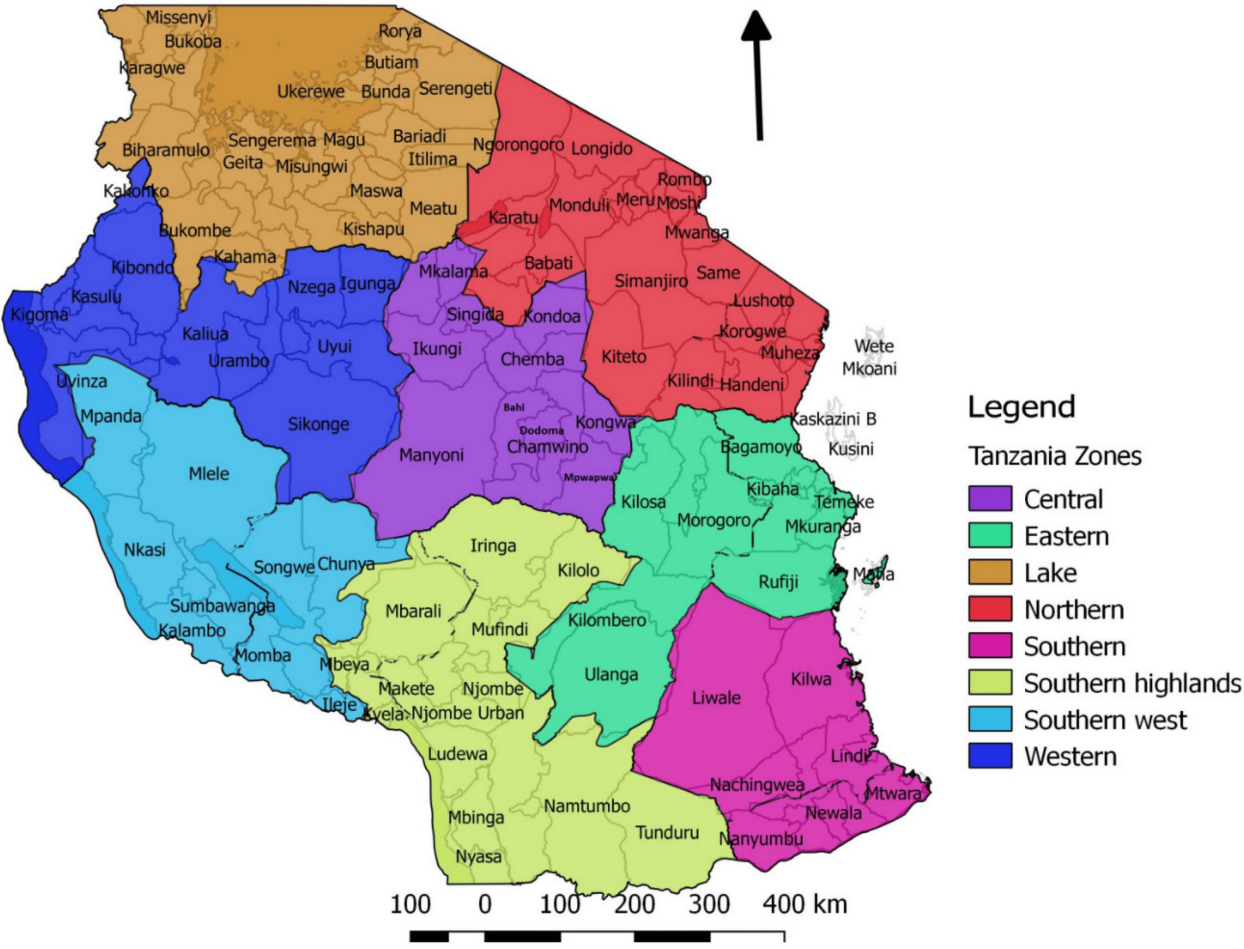
The distribution landscape of various administrative zones for STEMI service in Tanzania Permission obtained from the authors/Journal for the reuse of the figure [15] STEMI: ST-elevation myocardial infarction

**Figure 3:**
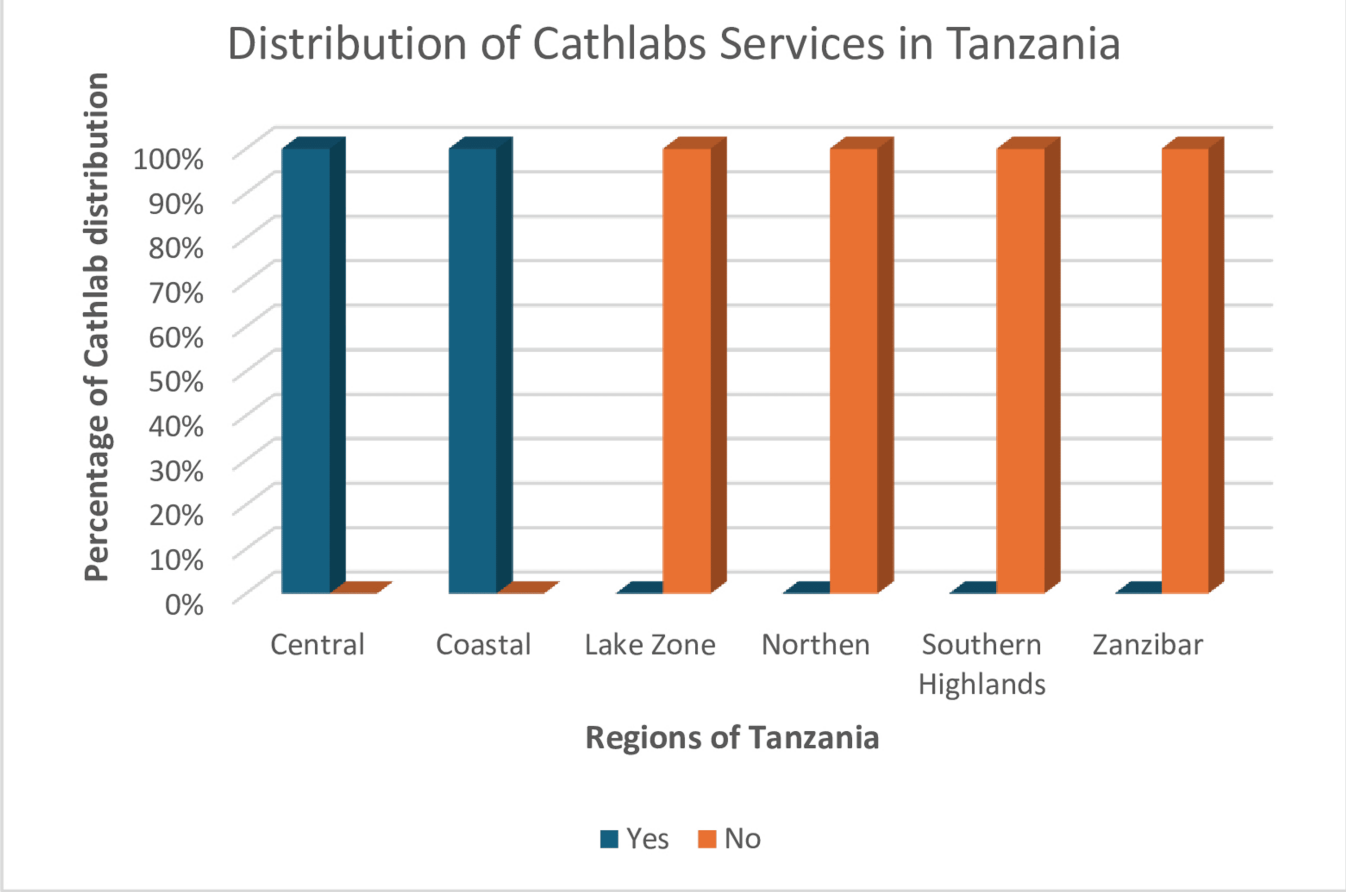
The distribution of cath labs service for STEMI in Tanzania STEMI: ST-elevation myocardial infarction

Primary angioplasty services were concentrated in the coastal and central regions of the country, with four catheterization laboratories in the Dar es Salaam region (coastal) and one in the Dodoma Region (central). The response rate was low for most centers (Figure 1). The country's distribution landscape based on various administrative zones is shown in Figure 2. Figure 3 shows the distribution of laboratory services for coronary angioplasty catheterization across the country.

As shown in Table 1, a highly statistically significant association was found between facility type and clinical responses in both private and public hospitals (χ²=29.91, p<0.001). This indicates that familiarity with thrombolysis pathways varied significantly among clinicians, depending on their employment facility (e.g., district, zonal, specialized hospitals).

Table 2 shows that the distribution of medical expertise and thrombolytic medicines for patients with STEMI differed between the public and private health systems. One-third (n=12, 33%) of patients in public facilities and (n=13, 43%) in private facilities were managed by general practitioners and internal medicine physicians, respectively; regarding thrombolytics in use, over three-quarters (27, 75%) of public facilities compared to just over half (16, 53%) of private facilities lacked this vital medicine. Public health has a slight advantage over private facilities in terms of most other medications required to manage STEMI. As shown in Table 2, there was no significant association between facility type (private vs. public) and any of the characteristics tested, including attending doctor, medicine availability, and thrombolysis type.

**Table 2:** Patient management and expertise of clinicians managing patients with acute coronary syndrome *Fisher’s exact test; Pearson’s Chi-squared test LMWH: low-molecular-weight heparin

Characteristic	Private (n=30), n (%)	Public (n=36), n (%)	P-value^*^	Chi-square value
Attending doctor			0.073	7.55
Cardiologist	6 (20)	4 (11)		
Clinical officer	1 (3.3)	1 (2.8)		
Emergency physician	8 (27)	9 (25)		
General practitioner	2 (6.7)	12 (33)		
Internal medicine physician	13 (43)	10 (28)		
Medicine availability				
Aspirin	21 (70)	26 (72)	0.8	0
Clopidogrel	21 (70)	28 (78)	0.5	0.19
LMWH	18 (60)	20 (56)	0.7	0.013
Statin	19 (63)	24 (67)	0.8	0.0006
Thrombolysis type			0.2	4.92
None	16 (53)	27 (75)		
Tenectaplase	8 (27)	3 (8.3)		
Streptokinase	3 (10)	4 (11)		
Ateplase	3 (10)	2 (5.6)		

Table 3 shows that the modality and duration of referral from one level to another were not significantly different between the private and public health facilities. However, it is worth noting that less than a quarter (23%) of private and nearly one-third (31%) of public health facilities had referral durations within the recommended limits of less than 90 minutes. There was no statistically significant association between referrals and facility type (χ²=3.76, p>0.05), as shown in Table 3. Similarly, there was no statistically significant association between referral duration and facility type (χ²=4.61, p>0.05).

**Table 3:** Referral methods and duration of referrals *Fisher’s exact test; Pearson’s Chi-squared test

Characteristic	Private (n=30), n (%)	Public (n=36), n (%)	P-value^*^	Chi-square value
Patient referral			0.2	3.76
Phone call	14 (47)	10 (29)		
Referral letter	16 (53)	24 (71)		
Unknown	0	2		
Referral duration, hours			0.3	4.61
>1.30 - ≤12	9 (30)	16 (44)		
>12 - ≤24	8 (27)	3 (8.3)		
>24 - ≤48	4 (13)	4 (11)		
>48	2 (6.7)	2 (5.6)		
≤1.30	7 (23)	11 (31)		

## Discussion

This survey, conducted in the second quarter of 2023, provides a glimpse of the general context regarding the infrastructure and medical expertise for managing patients with MI in Mainland Tanzania and Zanzibar. Primarily, the country's distribution of private and public facilities is uneven. The northern and coastal zones, which comprise nine out of 31 regions in the country, have more than two-thirds of all facilities that can manage patients with MI. Nearly all the facilities in the coastal zone are located in the Dar es Salaam region. This region has the largest commercial city in the country, accounting for 17.02% of the gross domestic product (GDP), which is higher than in most zones in the country [14]. This affects the accessibility and affordability of quality care. Low- and high-income countries exhibit similar patterns, and vulnerable populations, primarily residing in rural and peri-urban areas, are disproportionately affected [6,9,10,16,17].

In Tanzania, only a handful of facilities - primarily referral and tertiary hospitals - are equipped to manage patients with MI. Given the current statistics and dynamics, this is a pressing and significant challenge, as 84.7% of the facilities are dispensaries. Most of the population has access to and is mainly attended to in the primary healthcare sector, which falls into the category of dispensaries [14,16]. The highest age-standardized DALYs were recorded in relation to hypertensive heart disease in Sub-Saharan Africa, and IHD is on the rise. Patients with untreated conditions and those who receive delayed treatment are more likely to suffer from arrhythmias and heart failure, contributing to reduced quality of life, disability, and a significant burden on the economy of these countries. The STEMI pathway can reduce this by offering timely intervention to patients with a diagnosis of myocardial infarction [18].

The quality of primary healthcare in Tanzania, as in most LMICs, is limited in terms of expertise, medications, and infrastructure, even for common medical conditions [16,17,19,20]. This will have adverse effects on the outcomes of patients with MI, unless deliberate measures are implemented. Adequacy of skilled healthcare workers (HCWs) is vital for improving the quality of care. In our survey, only 20% and 11% of private and public health facilities, respectively, utilized cardiologists to manage patients with MI. The remaining facilities had lower-carded doctors for handling these patients. This is primarily due to the limited number of trained cardiologists and limited resources to run this service. Additionally, the number of centres providing coronary angioplasty is also small, and most of them are located in urban centres. A similar situation was observed regarding other LMICs. Africa, for example, accounts for about a quarter (24%) of the global disease burden. However, it has only 3% of global HCWs, and this continent, with a population of over 1.2 billion people, had only 2,000 cardiologists in 2019. Since then, the number has been estimated to have declined owing to COVID-19-related deaths and international migration [21,22]. 

Task-shifting, fostering a conducive working environment to encourage retention, and promoting cardiology training are critical for addressing this challenge. Patients with STEMI require timely and accurate diagnosis and management to preserve heart function. In resource-limited settings, fibrinolytics in combination with antiplatelets and anticoagulants are recommended as first-line treatment to stabilize patients before referring them promptly, within 90 minutes, for primary angioplasty. This survey revealed a significant shortage of fibrinolytics in both private and public facilities, with even fewer facilities capable of timely referral of patients for primary angioplasty within the guideline-recommended 24 hours if they had undergone thrombolysis and 90 minutes if they had not. This situation reflects a real-time scenario in other LMICs [3,13,16,22-24]. This is primarily due to a lack of resources, the unavailability of thrombolytic drugs, a shortage of expertise, and the absence of catheterization laboratories (cath labs) in most hospitals that can offer coronary angioplasties. More emphasis should be placed on distributing knowledge and skills for the prompt diagnosis and management of STEMI to improve STEMI networks. Stakeholders, such as the government through the Ministry of Health, play a significant role in subsidizing the cost of thrombolytics to ensure their availability. Investment by the government in building proper infrastructure would help reduce the travel time of referral patients to a primary angioplasty-capable center, thereby improving mortality and morbidity rates. These efforts will improve morbidity and enable Tanzania to achieve the Sustainable Development Goal target of reducing premature deaths due to NCDs by 25% by 2030 [2,12,25].

Strengths and limitations

Since the launch of the STEMI network in the country, this is the first survey to examine the availability of resources, including thrombolytic drugs, expertise, and the availability of cath labs for managing patients with acute STEMI. Therefore, its findings offer valuable insights into the general quality and capacity of facilities, providing a foundation for improvement, policy formulation, and further research. This was a cross-sectional study based on a pre-validated online survey; however, despite our best efforts, there is a risk of reporting and sampling bias due to the underrepresentation of specific centers. Consequently, it was not possible to establish a temporal association between the variables. Some facilities, such as polyclinics, have not joined the STEMI network. Furthermore, the survey did not involve taking an inventory or observation to ascertain the presence of expertise and medications. These findings must be interpreted in light of these gaps, and they provide a basis for further studies.

## Conclusions

Cardiovascular services in Tanzania are unevenly distributed, and their quality is questionable. Available expertise and medical infrastructure are inadequate, and there are slight to significant differences between private and public health facilities concerning available expertise, medications, referral systems, and the level at which most patients attend. In light of these findings, a proper needs assessment is required to inform appropriate policies and strategies for resource mobilization and allocation.
